# Annotations

**Published:** 1890-09-27

**Authors:** 


### ANNOTATIONS.

**Boy Suicides and Doctors.**

LAST week two boy suicides were reported from Vienna; this week another story of the same kind reaches us. The suicide mania has been prevalent for some time among the Vienna schoolboys. A youth of eighteen, the son of a Hungarian Judge, went up for examination at one of the military schools, where he had been taught for a year, and did not, as he thought, do well in mathematics. Fearing the result, he left the Academy without permission and went home to his family. He told them at dinner that he had left the Academy because he had failed in mathematics, and that he should choose some other career. After dinner he went to his room, and almost immediately the report of a revolver was heard. He was found dead on the floor. He had not, however, failed in the examination, but had passed, and been nominated for a higher class. He was a high-spirited, handsome youth; and his parents are beside themselves with grief at his death. This is not the case of a stupid boy, unfit for intellectual culture; it is the case of a clever and conscientious youth killed in his opening prime by the senseless mania for over-intellectual culture. In these days the tyranny of learning is driving the world to madness. Professors of all sorts have got the upper hand, and their supremacy threatens to be fatal to the rest of the world. Here is an opportunity for medicine. If doctors were large-minded and capable men like Shakespeare and Bacon, they would see the truth, and assert their scientific authority. But what are they doing? Nothing at all to guide the world in the matter! They are the worst offenders of all in the way of inflicting upon young men unlimited intellectual tasks, the doing of which is of no practical service to anybody. Sleeplessness, nervousness, mania in every form are upon us, and nothing is done. The whole medical world itself is in full cry striving who shall be first to put salt on the tail of the tubercle bacillus. It is as if all the forces of the empire should be sent to arrest a lunatic at Wick whilst a foreign army was in possession of Penzance. We are a profession of grubbers. The infinitely little alone has any charms for us. The great we cannot deal with at all. The future of the human race depends upon the sound bodily and mental health of the civilised peoples. We are permitting schoolmasters and examiners to ruin both without a word of protest.

**No Gentleman Keeps a Bull-dog.**

FRANK SLAVIN and Joe McAuliffe were brought before Mr. Partridge at the Lambeth Police-court on Monday, charged with having agreed to commit a breach of the peace by engaging to take part in a prize fight at the Ormonde Club, Walworth Road. The court was crowded to its utmost capacity with people anxious to hear the case, and large numbers of curious sympathisers were unable to obtain admission. Darwin has taught us the inestimable value of looking at everything dispassionately. By so doing we can generally put things into their right places, and deal with them as they ought to be dealt with, without unnecessary worry or fuss. The religious man wrings his hands in despair at what he considers the brutality of the prizefighter and his friends. The man of culture and breeding, the gentleman, in short, can find no words to express his contempt and disgust. But there is another aspect of the question. It is as plain as possible that certain animals which are destitute of what we call intellect have a love of fighting, a savage and determined taste for blood and murder. The bull-dog, for example, is not a mere beast of prey, who catches wild creatures because he is hungry: he is a typical example of unmitigated savagery and blood-lust. We cannot help sickening a little when we witness a fight between two bull dogs. The sight is quite enough to make any man resolve not to keep a bull-dog on any account; and since there is no longer any use for such animals in this country every gentleman in the land would be very willing to decree their prompt extinction. No gentleman keeps a bull-dog. The man whose tastes lie in that direction cannot by the laws of science be a gentleman. He must necessarily have an undue prominence of the brute in his composition. What has been said of the bull-dog and his lovers may be said with ten-fold truth of the prize fighter and his admirers. Science compels us to classify creatures according to their most pronounced and obvious similarities. Prize fighters and those who run after them have the bodies and limbs of human beings, and some superficial resemblance to men in their faces and features. But in order to make clear their place in the animal creation it is only necessary to turn them loose in a convenient spot. The principals will at once begin to try their best to kill each other in cold blood; the onlookers will reveal their savage-beast nature still more clearly by their inhuman howls, and grins, and gestures. Men scientifically educated are beginning to claim a wider range of authority over the animal world than their forefathers did. There are many who think that poor-brained and feebly-developed men and women should be prevented by civilised states from marrying; there are others who hold that hereditary and incorrigible criminals should be destroyed in the interests of the State. If this high scientific view should ultimately prevail, it is certain that prize-fighters and their admirers will be included among those whom the State will doom to extirpation. The question need not be discussed from a moral point of view at all; bull dogs have no morals, neither have prize-fighters and their friends. So far as they are of any value to society, both the dogs and the prize-fighters might all be knocked on the head like hungry wolves, without further consideration.

**Cardinal Lavigerie and Man Hunting.**

THOSE who have been in certain parts of Africa or who have friends there are very familiar with the fact that man hunting is an occupation whereby men live and grow rich exactly the same as elephant hunting or cattle raising. Bands of Arabs, with their leaders, attack villages and small settlements of natives, and kill as many as are necessary to make the others submit to be captured. The whole of the remaining villagers, men, women, and children, are then driven through the forests to the nearest slave market, and there sold to the highest bidder like ordinary cattle. Many of the bidders are foreigners, who buy and ship their human "stock" to distant countries and there re-sell them to those who want them for work or for worse purposes. Some people think, or affect to think, that the native Africans have so little intellect that they do not feel the horrors of slavery as we do. There is no more truth in that than there is in the statement that a hungry child does not suffer distress by being deprived of its food. The real want of intellect is not among the natives, but among the selfish and brainless creatures who put forward such unworthy theories. The writer himself knows a young man well, who, when he was a boy of thirteen, was in a village that was attacked by slave raiders. The boy, rather than fall into their hands, rushed to the woods, and set out, all alone, through the trackless African jungle for the nearest mission station. He had no idea how far distant it might be; he only knew the general direction in which it lay. For six weeks he braved hunger, homelessness, the tropical sun, and the wild beasts of the wood; living on roots and berries in the daytime, and sleeping in the trees at night like a monkey. He found the mission at last, and with it his freedom. Had such a boy no just apprehension of the horrors and miseries of slavery? Cardinal Lavigerie would fain put an end to this man-hunting and village-destroying; and if no other way can be found, he recommends the shooting of the man-hunters. Thereupon the *Daily News* takes him to task for advocating methods inconsistent with Christianity. This is sheer nonsense. Is it inconsistent with Christianity to hang a murderer? It is no more inconsistent to shoot down a ruffian who has probably already killed at least a dozen blacks in his nefarious trade, and is prepared to kill a thousand more if necessary. The chief of all the things that Christianity teaches in regard to mutual duty is that men should do as they would be done by. If we ourselves were Africans in African villages, should we or should we not wish our lives, our liberties, the safety of our children, and the honour of our wives and daughters, to be defended, even at the cost of the lives of the assailants? Most assuredly we should. And the advice we give to the militant Cardinal is that if he can raise and maintain a sufficient force to make his shooting entirely successful, the sooner he begins his Christian work the better. The only thing that we can see which would be unchristian in such a campaign would be the setting out with an insufficient force. Christianity is at least as much a religion of working and fighting as of praying and preaching.

